# Family Smoking, Exposure to Secondhand Smoke at Home and Family Unhappiness in Children

**DOI:** 10.3390/ijerph121114557

**Published:** 2015-11-13

**Authors:** Jian Jiu Chen, Sai Yin Ho, Wing Man Au, Man Ping Wang, Tai Hing Lam

**Affiliations:** 1 School of Public Health, University of Hong Kong, Hong Kong, China; E-Mails: chenjianjiu@gmail.com (J.J.C.); awm_anson1210@hotmail.com (W.M.A.); hrmrlth@hku.hk (T.H.L.); 2 School of Nursing, University of Hong Kong, Hong Kong, China; E-Mail: mpwang@hku.hk

**Keywords:** happiness, secondhand smoke, family, children

## Abstract

Tobacco use adversely affects many aspects of well-being and is disliked by non-smokers. However, its association with family happiness is unknown. We investigated the associations of family unhappiness with smoking in family members and secondhand smoke (SHS) exposure at home in Hong Kong children. In a school-based survey in 2012–2013, 1238 primary school students (mean age 8.5 years, standard deviation 0.9; 42.6% boys) reported family smoking, SHS exposure at home and whether their families had any unpleasant experience caused by smoking or SHS in the past 30 days (tobacco-related unpleasant experience), and rated the overall level of happiness in their families (family unhappiness). Multivariable logistic regression was used to study the associations of tobacco-related unpleasant experience and family unhappiness with family smoking and SHS exposure at home. Tobacco-related unpleasant experience and family unhappiness were reported by 27.5% and 16.5% of students. Unpleasant experience was more strongly associated with family smoking than SHS exposure at home. Family unhappiness was associated with both family smoking (odds ratio 2.37; 95% confidence interval 1.51–3.71) and SHS exposure at home (1.82; 1.39–2.40). These results suggest a previously neglected possible impact of tobacco use on family happiness.

## 1. Introduction

Smoking [1] and exposure to secondhand smoke (SHS) [2] are both major hazards to physical health. The prospective associations of smoking with depression [3,4,5,6,7,8,9] and anxiety [10,11,12,13,14,15] are well established, and a causal interpretation is underpinned by biological [16,17,18,19,20] and psychosocial mechanisms [3]. SHS has also recently been associated with mental disorders on cross-sectional [21,22,23,24,25,26] and longitudinal levels [26,27].

Recent research suggests an impact of smoking on happiness. In a cross-sectional survey of ex-smokers in the United Kingdom (UK), 69.3% reported feeling happier now than when they were smokers [28]. In two cross-sectional studies, one in England [29] and the other in nine former Soviet Union (fSU) countries [30], ex-smokers and those who had never smoked were both happier than current smokers. In a cross-sectional study in Hong Kong, ex-smokers were happier than those who had never smoked, yet those who had never smoked and current smokers were similar [31]. A study in the United States (US) recruited a sample of smoking parents and found 12 months after recruitment that the parents who quit smoking were happier than those who did not quit [32]. An opposite finding, *i.e.*, smokers being happier than non-smokers, was reported in Chilean college students [33], and a null finding was reported in Japanese workers [34].

Research suggests that non-smokers’ perceptions of SHS and family members’ smoking are generally negative, although such research is surprisingly limited. In a survey in US preteens, when asked about the reactions towards SHS, 76.6%, 56.6%, 24.1% and 21.7% reported “unpleasant or gross”, “coughed or chocked”, “dizzy” and “wanted to throw up”, respectively [35]. In a survey of adolescents in Scotland, 93.6% strongly disapproved of people smoking near them [36]. Other surveys suggest similar dislike of SHS [37,38]. Moreover, a cross-sectional study of Iranian adolescent girls found that SHS was associated with unhappiness [39]. Qualitative studies in children [40], adolescents [41] and adults [42] have consistently showed strong negative feelings towards family members’ smoking and SHS. While the negative feelings in children and adolescents were largely due to the health concerns for smoking family members and themselves as well as the physical discomforts induced by SHS, such as coughing and eye irritation [40,41], the negative feelings in adults were additionally due to the expenditure on buying cigarettes [42].

Given the various negative impacts of tobacco use and the dislike of tobacco use in non-smokers, it is possible for smoking and SHS to lead to unhappiness in a family. However, we found no such studies in the literature. Family happiness is highly valued in many cultures and may have important implications for children. Children from unhappy families tend to have poorer personal and social adjustment in later life [43]. Therefore, the present study aimed to investigate the associations of smoking in family members and SHS exposure at home with family unhappiness using cross-sectional data from primary school students in Hong Kong.

## 2. Methods

### 2.1. Sampling and Ethics Statement

Each year, all primary schools in Hong Kong (about 500) received invitation to enroll for an educational theatre stage performance organised by the Hong Kong Council on Smoking and Health, and the first 99 schools to respond were accepted. In 2012–2013, 10 schools were randomly selected from the 99 accepted schools to be invited to participate in a cross-sectional survey for students in Grade 2–4 (equivalent to Grades 2–4 in the US). Invitation letters were sent to parents via students. Declining parents were to ask their child to return a blank questionnaire after the survey. Student participation was voluntary even with parental permission. Ethical approval was obtained from the Institutional Review Board of the University of Hong Kong/Hospital Authority Hong Kong West Cluster.

### 2.2. Measurement

An anonymous and self-administered questionnaire in simple Chinese was used. Two questions were related to family happiness: (1) “In the past 30 days, was there any unpleasant experience caused by smoking or secondhand smoke in your family?” (tobacco-related unpleasant experience) with response options of no, seldom, sometimes and often; (2) “In general, you think your family is:” (family unhappiness) with response options of very unhappy, unhappy, happy and very happy.

SHS exposure at home was measured by one question: “How many days in the past 7 days did someone smoke near you at home?” Response options of 0 to 7 days/week were grouped into 2 (none, any) and 3 (none, 1–3 days/week, 4–7 days/week) levels. The number of co-residing smokers (family smoking) was also grouped into 2 (none, any) and 3 (none, 1 smoker, 2 or more smokers) levels.

Students also reported their age (≤7/8/9/10/11/≥12), sex (male/female), perceived family affluence (rather poor/medium low/medium/medium high/rather wealthy), number of bedrooms at home (0/1/2/3/4/≥5), SHS exposure outside home (0/1/2/3/4/5/6/7 days/week) and biological parents’ marital status (married/divorced/father deceased/mother deceased/others). Marital status of biological parents’ was recoded as “married” and “others” because the proportion of students choosing items other than married was small. Number of bedrooms was used as a proxy indicator of socioeconomic status (SES) because living area is regarded as a good indicator of family SES in Hong Kong, where housing price is highest in the world [44].

### 2.3. Statistical Analysis

Ordinal logistic regression was considered for studying the associations between the study factors (family smoking and SHS at home) and the outcome variables (tobacco-related unpleasant experience and family unhappiness) but was not used because the proportional odds assumption was violated for both of the outcome variables (*p* < 0.001). Logistic regression was thus used with family unhappiness dichotomised as “happy” (very happy/happy) and “unhappy” (unhappy/ very unhappy) and tobacco-related unpleasant experience dichotomised as “no” and “any” (seldom/sometimes/often).

To study the associations of family smoking (study factor) with tobacco-related unpleasant experience and family unhappiness (outcome variables), multivariable logistic regression was used, adjusting for age, sex, perceived family affluence and marital status of biological parents and school clustering effect (Model 1). To further study the above associations independent of SHS at home, the above analyses were conducted again after excluding students with SHS at home (Model 2). To study the associations of SHS at home (study factor) with the outcome variables independent of family smoking, the students without co-residing smokers were excluded and multivariable logistic regression was used, adjusting for the number of co-residing smokers at home (1/2/3/4/5 or more) in addition to the same set of covariates adjusted in the previous model and school clustering (Model 3). In the above analyses, the study factors (family smoking and SHS at home) were analysed as binary and 3-level variables, and the dose-response relationships were studied by treating the 3-level study factors as continuous variables. Exclusion was used in the above analyses for the following reasons. The associations of interest in the excluded and remained students might be theoretically different (e.g., the associations of SHS at home with the outcome variables in students with and without family smoking might be different). The potential interaction effect was thus tested using an interaction term of SHS at home * family smoking and significant *p* values were found for interaction between 5–7 days/week of SHS at home (*vs.* 0 days/week) and 2 or more co-residing smokers (*vs.* no co-residing smoker) for both outcome variables (*p* < 0.05). Such interaction effects suggested stratification of analyses. However, an exclusion was used because the situation in which students were exposed to SHS at home but with no family smoking was rare (n = 19, 1.5% of the sample), making the associations of interest in the excluded students (strata) less relevant to real life and their estimates susceptible to random variation.

It is worth noting that tobacco-related unpleasant experience is, by definition, caused by smoking or SHS. The purpose of studying its associations with family smoking and SHS was to compare the relative importance of these 2 study factors.

The reliability of the variables related to family happiness and SHS was assessed in a test-retest study with an interval of 8 days in 298 Grade 2–4 students in another primary school (boys 52.4%; mean age 8.9, standard deviation 1.1 years). The intra-class correlation coefficients were 0.82, 0.71 and 0.88 for tobacco-related unpleasant experience, family unhappiness and SHS exposure, respectively; the intra-class correlation coefficient was 0.86 for SHS exposure when 3-level responses were compared; and percentage agreement (comparing dichotomized responses) were 87.3%, 84.2% and 86.0%, respectively. These results indicated good test-retest reliability.

A series of sensitivity analyses were conducted. Firstly, to examine whether the results were robust when the outcomes were analysed as 4-level instead of binary variables, the main analysis was conducted again using multi-nominal logistic regression and setting “very happy” as the reference level for family unhappiness and “no” as the reference level for tobacco-related unpleasant experience.

Secondly, since complete case analysis was used, the Model 1 for tobacco-related unpleasant experience and family unhappiness used 1075 and 1091 cases, accounting for 86.8% and 88.1% of the cases remained for analysis (1238). Although such proportions of missingness were unlikely to induce severe bias, multiple imputation was conducted for the analysis for family unhappiness, which was the more important outcome variable. The imputation model incorporated both of the outcome variables, both of the study factors, the covariates in the main analysis and number of bedrooms at home.

Thirdly, we added a step of coarsened exact matching (CEM) before the multivariable regression and after the exclusion, if any, in the main analysis. This approach is considered to produce estimates that are less model dependent, more accurate and have less potential for confounding bias [45,46]. A description of the sensitivity analyses is shown in the Supplementary Files.

All analyses were conducted in STATA 13.0.

## 3. Results

Of the 10 randomly selected schools, seven agreed to participate in the survey. The seven schools had 1367 students in Grade 2–4, and 1255 students (91.8%) returned a valid questionnaire. After excluding 17 (1%) questionnaires with inconsistent information, 1238 questionnaires remained for analysis.

Table 1 shows that 42.6% of subjects were boys and the mean age (standard deviation) was 8.5 (0.9) years. The proportions of students reporting tobacco-related unpleasant experience was 27.5% overall and 48% (shown in Table 2) in those with at least one co-residing smoker. Family unhappiness was reported by 16.5% of students. The prevalence of family smoking and SHS exposure at home was 41.0% and 24.0%, respectively.

**Table 1:** Characteristics of Grade 2–4 students in Hong Kong.

Characteristics	N	% ^a^
Sex		
Boys	507	42.6
Girls	682	57.4
Age		
≤7 to 8	667	54.3
9 to ≥12	562	45.7
Mean age (*standard deviation*) in years	1229	8.5 (0.9)
Number of bedrooms at home		
1 or no	160	13.2
2	597	49.3
3 or more	453	37.4
Perceived family affluence		
Poor and poor to medium	150	12.4
Medium	657	54.2
Medium to rich and rich	405	33.4
Marital status of biological parents		
Married	1007	84.3
Others	188	15.7
Number of co-residing smokers		
None	724	59.1
1	373	30.4
2 or more	129	10.5
Any	502	41.0
SHS at home		
None	912	76.0
1–4 days/week	145	12.1
5–7 days/week	143	11.9
Any	288	24.0
SHS outside home		
None	672	55.5
1–4 days/week	352	29.0
5–7 days/week	188	15.5
Any	540	44.5
Tobacco-related unpleasant experience in the past 30 days		
No	853	72.5
Yes	323	27.5
Family unhappiness		
No	1000	83.5
Yes	197	16.5

Note: ^a^ Proportion of students unless otherwise stated.

Table 2 shows that among students without SHS at home, tobacco-related unpleasant experience was significantly and strongly associated with family smoking. In contrast, among students with co-residing smokers, the associations between tobacco-related unpleasant experience and SHS at home were apparently weaker and either marginally significant or non-significant.

**Table 2:** Adjusted odds ratio (AOR) of tobacco-related unpleasant experience by family smoking and secondhand smoke (SHS) exposure at home in primary students in Hong Kong.

**Study Factors**	**Model 1**		**Model 2**	
	**N = 1075 ^a^**		**N = 836 ^c^**	
Number of co-residing smokers at home	Unpleasant experience (%)	AOR (95% CI) ^b^	Unpleasant experience (%)	AOR (95% CI) ^d^
None	13.6	1	13.5	1
1	46.9	5.93 (4.36–8.06) ***	41.6	4.64 (3.55–6.06) ***
2 or more	51.2	6.74 (5.06–8.97) ***	46.9	6.60 (3.53–12.33) ***
Any	48.0	6.11 (4.82–7.75) ***	42.8	4.97 (4.19–5.89) ***
*p* for trend		<0.001		<0.001
**Study Factors**			**Model 3**	
			**N = 421 ^e^**	
SHS exposure at home			Unpleasant experience (%)	AOR (95% CI) ^f^
None			41.2	1
1–4 days/week			46.3	1.15 (1.01–1.32) *
5–7 days/week			58.9	1.77 (0.82–3.83)
Any			52.8	1.43 (0.98–2.07)
*p* for trend				0.13

Note: * *p* < 0.05; *** *p* < 0.001; ^a^ Complete case analysis; ^b^ Adjusting for age, sex, perceived family affluence, marital status of biological parents and school clustering; ^c^ Complete case analysis and excluding students with SHS at home; ^d^ Adjusting for age, sex, perceived family affluence, marital status of biological parents and school clustering; ^e^ Complete case analysis and excluding students without family smoking; ^f^ Adjusting for the number of co-residing smokers at home, age, sex, perceived family affluence, marital status of biological parents and school clustering.

Table 3 shows that any co-residing smoker was associated with an adjusted odds ratio (AOR) (95% confidence interval (CI)) of 2.99 (1.96–4.54) for family unhappiness, compared with no co-residing smoker. The corresponding AORs for one and two or more co-residing smokers were 3.01 (2.10–4.32) and 2.90 (1.39–6.06), respectively (*p* for trend < 0.001). When restricting the analysis to students without SHS at home, such associations remained statistically significant. Among students with co-residing smokers, any SHS at home was associated with an AOR of 1.82 (1.39–2.40) for family unhappiness, compared with no SHS at home. The corresponding AORs for 1–4 and 5–7 days/week of SHS at home were 1.53 (1.12–2.09) and 2.17 (1.35–3.50), respectively (*p* for trend = 0.001).

**Table 3:** Adjusted odds ratio (AOR) of family unhappiness by family smoking and SHS exposure at home in primary students in Hong Kong.

**Study factors**	**Model 1**		**Model 2**	
	**N = 1091 ^a^**		**N = 844 ^c^**	
Number of co-residing smokers at home	Family unhappiness (%)	AOR (95% CI) ^b^	Family unhappiness (%)	AOR (95% CI) ^d^
None	8.8	1	8.8	1
1	25.5	3.01 (2.10–4.32) ***	20.1	2.10 (1.38–3.20) **
2 or more	27.9	2.90 (1.39–6.06) **	30.8	3.66 (1.28–10.46) *
Any	26.1	2.99 (1.96–4.54) ***	22.6	2.37 (1.51–3.71) ***
*p* for trend		<0.001		0.001
**Study Factors**			**Model 3**	
			**N = 429 ^e^**	
SHS exposure at home			Family unhappiness (%)	AOR (95% CI) ^f^
None			20.7	1
1–4 days/week			26.8	1.53 (1.12–2.09) **
5–7 days/week			35.3	2.17 (1.35–3.50) **
Any			31.1	1.82 (1.39–2.40) ***
*p* for trend				0.001

Notes: * *p* < 0.05; ** *p* < 0.01; *** *p* < 0.001; ^a^ Complete case analysis; ^b^ Adjusting for age, sex, perceived family affluence, marital status of biological parents and school clustering; ^c^ Complete case analysis and excluding students with SHS at home; ^d^ Adjusting for age, sex, perceived family affluence, marital status of biological parents and school clustering; ^e^ Complete case analysis and excluding students without family smoking; ^f^ Adjusting for the number of co-residing smokers at home, age, sex, perceived family affluence, marital status of biological parents and school clustering.

The results of the multi-nominal logistic regression (Tables S1 and S2 in the Supplementary Files), multiple imputation (Table S3 in the Supplementary Files) and CEM (Tables S4 and S5 in the Supplementary Files) were not meaningfully different from the main analyses (Table 2 and Table 3).

## 4. Discussion

Our study found that family smoking, SHS exposure at home, and tobacco-related unpleasant experience were prevalent in Hong Kong families. In particular, half of the families with one or more smokers had tobacco-related unpleasant experience in the past 30 days. Such unpleasant experience was more strongly associated with family smoking than SHS, suggesting that, to avoid the unpleasant experience, merely avoiding smoking at home is insufficient, and quitting smoking is needed.

Our study found that family smoking was associated with family unhappiness. Such associations remained statistically significant among students without SHS at home, which indicated that having co-residing smokers who did not smoke at home was associated with increased family unhappiness compared with having neither co-residing smoker nor SHS at home. We also found that SHS at home was associated with family unhappiness among students with co-residing smoker(s), which indicated that, for students who had co-residing smoker(s), no SHS at home was associated with increased family happiness. All the observed associations were robust in the sensitivity analyses.

Family smoking and SHS may lead to dissatisfaction in non-smoking family members for many reasons. Apart from the physical discomforts caused by SHS, such as coughing and eye irritation, dissatisfaction in passive smokers may spring from the health concerns for themselves and their smoking family members. Such health concerns have been consistently documented in qualitative studies in non-smokers of various age groups and socio-economic status [40,41,42]. Furthermore, a qualitative study in the UK showed that adolescents challenged their parents’ smoking, expressed disgust and concern and hid or destroyed cigarettes [41]. Such resistance also reflected the adolescents’ dissatisfaction and health concerns related to tobacco. In Hong Kong, the dissatisfaction due to health concern may be particularly strong, because of the widespread health education on the harm of tobacco, the stringent tobacco control legislation and the smoking prevalence (10.7% in 2012) that is among the lowest in the world [47]. The dissatisfaction could be exacerbated by the financial burden of buying cigarettes. It is well established in population studies that expenditure on cigarettes crowds out the expenditure on other household essentials, such as food, housing and clothing [48,49,50,51]. A non-smoking adult subject said in a qualitative study: “How can they (smokers) use money for cigarettes? It’s like taking food off the table.” [42]. In Hong Kong, the expenditure on cigarettes can be substantial. Given that a pack of cigarettes costs about 54 Hong Kong (HK) dollars (US$ 7.0), a smoker consuming one pack per day would spend HK$19710 per year on cigarettes, nearly the median monthly household income of HK$22,400 in 2013 [52]. Such avoidable expenditure that brings harm may easily displease non-smoking family members. In the present study, the high prevalence of tobacco-related unpleasant experience is a clear indication of the dissatisfaction in the non-smoking family members. The dissatisfaction may in turn undermine family happiness.

To our knowledge, this is the first study to report associations of family smoking and SHS exposure at home with family unhappiness. The newly discovered associations, however, should be interpreted with caution because of the cross-sectional design. However, given the dissatisfaction in non-smokers, which could be compounded by multiple reasons, and the high prevalence of tobacco-related unpleasant experience, these associations suggest a previously neglected impact of tobacco use on interpersonal well-being and family happiness and also provide a justification for such impact to be further explored in future research.

The impact of tobacco use on family happiness, if confirmed in future research, shall have important implications. It expands the scope of the evaluation of the impact of tobacco use, adds to the justification for stronger tobacco control measures and suggests a new message for health education that focuses on interpersonal well-being and family happiness as the immediate benefits of smoking cessation and avoiding SHS. Interestingly, a study in Norway found that the introduction of a smoking ban in bars and restaurants increased the job satisfaction in non-smoking employees [53].

The present study has several limitations. First, residual confounding might exist given the limited number of potential confounders in our model. Nonetheless, we addressed this issue by controlling for all the covariates in our dataset (except those that could be affected by the study factors or outcomes) using CEM combined with adjustment in multivariable regression. Second, temporality between family smoking, SHS and family unhappiness cannot be ascertained because of the cross-sectional design. However, smoking initiation should generally precede marriage, because most smokers started smoking in adolescence or early adulthood. In addition, whether smokers avoid smoking at home may more likely to be determined by factors other than family happiness. Third, the two study factors (family smoking and SHS at home) were self-reported and thus subject to misclassification. However, the smoking behaviour of co-residing family members should be obvious to children, especially in Hong Kong, where homes are typically small. Self-reported SHS at home are needed in this study because biomarkers of SHS exposure cannot distinguish exposure from home and other places. Moreover, self-reported SHS exposure by children has been validated using hair nicotine levels in our previous study [54] and its test-retest reliability has been shown to be good in the present study. Any random misclassification of the study factors would have biased the associations towards null. Fourth, in addition to the two study factors, thirdhand smoke (THS) exposure at home may also contribute to unpleasant experience and family unhappiness, given the generally negative self-reported reactions to THS in children [55]. Since THS should also be associated with the study factors, it may have influenced our estimates. For example, among the students with co-residing smoker(s), the lower level of family happiness in the group exposed to SHS at home than the group not exposed might partly be due to THS, because while the former group almost surely had THS exposure at home, only part of the latter group had such exposure (some co-residing smokers may smoke at home when children are not around, resulting in THS exposure). Future research should explore the association of family unhappiness with environmental tobacco smoke, including both SHS and THS [56], and how SHS and THS contribute to this association. Fifth, although the two outcome variables on family unhappiness reported by children showed good test-retest reliability, they only represent family happiness perceived by children and may not necessarily be the same with the perception of other family members. Nonetheless, children’s perception of family unhappiness is a meaningful outcome because it should be more directly relevant with the well-being and development of the children. Finally, these results from Hong Kong may not be generalisable to other areas. The dissatisfaction towards tobacco use may be stronger in areas where smoking is highly socially unacceptable.

## 5. Conclusions

Family smoking, SHS exposure at home and tobacco-related unpleasant experiences were prevalent in Hong Kong families. Family unhappiness reported by children was associated with both family smoking and SHS exposure at home. These results suggest a previously neglected impact of tobacco use on family happiness.

## Supplementary Materials

Supplementary File 1
