# Supplementary material for: Family Smoking, Exposure to Secondhand Smoke at Home and Family Unhappiness in Children
Source: Int J Environ Res Public Health. 2015 Nov 13;12(11):14557–70. doi: 10.3390/ijerph121114557 (PMC4661667; doi:10.3390/ijerph121114557)
Supplement: Supplementary File 1 [file ijerph-12-14557-s001.pdf]

Supplementary Information

Family Smoking, Exposure to Secondhand Smoke at Home and Family Unhappiness in Children

Coarsened exact matching (CEM) is a recently proposed matching approach that reduces imbalance in covariates between exposed and unexposed groups by temporarily coarsening the values of covariates, exact matching on the coarsened values, thereby generating a subset of matched data, and conducting analysis on the matched data using initial (uncoarsened) values of the covariates [1,2]. The coarsening of the values of covariates means to recode the values into broader categories. For example, age of ≤7/8/9/10/11/≥12 can be coarsened as ≤7–8/9–≥12. Exact matching on the coarsened values results in a subset of the original data because the matching procedure prunes the observations in one group (exposed or unexposed), with which no matched observations can be found in the other group.

CEM has several attractive properties such as using monotonic imbalance bounding (so that improving the balance on one variable does not affect the others), automatically restricting to common empirical support and balancing interactions between groups [1,2]. Moreover, CEM can be applied to multi-category treatment without further modification [2]. The multivariable regression is still needed after matching because unless the matching is exact, the regression adjustment of covariates still have the potential to reduce confounding bias [1–3].

In the analyses where family smoking was used as the study factor, the covariates selected for CEM and their cut-offs, by which coarsening was performed, were as follows: age (≤7–8/9–≥12), number of bedrooms at home (0–1/2/3–≥5), perceived family affluence (rather poor to medium low/medium/medium high to rather wealthy) and SHS (SHS) exposure outside home (days per week) (0/1–4/5–7). Sex and marital status of biological parents were selected for matching but were not coarsened. The multivariable regression analyses followed adjusted for the initial values of the covariates that were coarsened (*i.e.*, age, number of bedrooms at home, perceived family affluence and SHS outside home) and school clustering. In the analyses where SHS at home was used as the study factor, the number of co-residing smokers at home was selected for CEM (cut-off: 1/2 or more), in addition to the same set of covariates as above. Similarly, the multivariable regression followed adjusted for the initial values of the covariates that were coarsened and school clustering.

The selection of covariates for CEM should be based on the consideration of the trade-off between the bias of excluding relevant covariates and the inefficiency of including irrelevant ones [3]. In the present study, however, the major concern was the possible confounding bias rather than the increased variance due to loss of data. For this reason, we selected all the available variables in our dataset as covariates, except those that could be affected by the study factors or outcomes.

Table S1. Adjusted odds ratio (AOR) of tobacco-related unpleasant experience by family smoking and SHS exposure at home (by multi-nominal logistic regression).

**Study Factors**	**Model 1, N = 1075 [a] Unpleasant Experience, AOR (95% CI) [b]**				**Model 2, N = 836 [c] Unpleasant Experience, AOR (95% CI) [d]**			
Number of co-residing smokers at home	No	Seldom	Sometimes	Often	No	Seldom	Sometimes	Often
None	1	1	1	1	1	1	1	1
1	1	6.70 (4.24–10.14) ***	6.09 (4.29–8.64) ***	4.82 (2.78–8.34) ***	1	4.73 (3.14–7.11) ***	4.19 (2.70–6.50) ***	5.08 (3.16–8.16) ***
2 or more	1	4.80 (2.97–7.78) ***	7.49 (4.02–13.97) ***	7.87 (4.95–12.52) ***	1	5.50 (2.18–13.87) ***	7.11 (4.10–12.33) ***	7.41 (2.04–26.90) **
Any	1	6.26 (4.28–9.17) ***	6.41 (4.33–9.49) ***	5.56 (3.87–7.98) ***	1	4.86 (3.31–7.12) ***	4.61 (3.13–6.81) ***	5.53 (3.23–9.47) ***
p for trend		<0.001	<0.001	<0.001		<0.001	<0.001	<0.001
					**Model 3, N= 421 [e] Unpleasant Experience, AOR (95% CI) [f]**			
SHS exposure at home					No	Seldom	Sometimes	Often
None					1	1	1	1
1–4 days/week					1	1.44 (1.05-1.97) *	1.37 (1.06–1.77) *	0.50 (0.08–3.14)
5–7 days/week					1	1.31 (0.47–3.67)	1.61 (0.86-3.02)	2.67 (1.10–6.46) *
Any					1	1.39 (0.78–2.48)	1.48 (1.14–1.91) **	1.46 (0.79–2.70)
p for trend						0.51	0.09	0.05

Notes: * $p < 0.05$; ** $p < 0.01$; *** $p < 0.001$; [a] Complete case analysis; [b] Adjusting for age, sex, perceived family affluence, marital status of biological parents and school clustering; [c] Complete case analysis and excluding students with SHS at home; [d] Adjusting for age, sex, perceived family affluence, marital status of biological parents and school clustering; [e] Complete case analysis and excluding students without family smoking; [f] Adjusting for the number of co-residing smokers at home, age, sex, perceived family affluence, marital status of biological parents and school clustering.

Table S2. Adjusted odds ratio (AOR) of family unhappiness by family smoking and SHS exposure at home (by multi-nominal logistic regression).

Study Factors	Model 1, N = 1091 [a] Family Unhappiness, AOR (95% CI) [b]				Model 2, N = 844 [c] Family Unhappiness, AOR (95% CI) [d]			
Number of co-residing smokers at home	Very happy	Happy	Unhappy	Very unhappy	Very happy	Happy	Unhappy	Very unhappy
None	1	1	1	1	1	1	1	1
1	1	2.25 (1.81–2.79) ***	7.68 (4.87–-12.11) ***	2.37 (1.14–4.91) *	1	2.13 (1.52–2.98) ***	5.86 (3.02–11.37) ***	1.18 (0.39–3.58)
2 or more	1	2.45 (1.81–3.31) ***	6.69 (2.89–15.53) ***	3.15 (1.30–7.59) *	1	1.39 (0.58–3.35)	5.10 (1.20–21.68) *	3.58 (1.07–11.96) *
Any	1	2.29 (1.88–2.80) ***	7.46 (4.73–11.78) ***	2.56 (1.36–4.80) **	1	1.97 (1.42–2.73) ***	5.69 (3.12–10.39) ***	1.71 (0.89–3.27)
p for trend		<0.001	<0.001	0.002		0.003	<0.001	0.04
					Model 3, N = 429 [e] Family Unhappiness, AOR (95% CI) [f]			
SHS exposure at home					Very happy	Happy	Unhappy	Very unhappy
None					1	1	1	1
1–4 days/week					1	1.13 (0.83–1.54)	1.58 (0.94–2.66)	1.80 (0.82–3.91)
5–7 days/week					1	1.54 (0.95–2.50)	2.55 (1.54–4.24) ***	3.81 (1.80–8.06) ***
Any					1	1.31 (0.95–1.79)	1.98 (1.36–2.90) ***	2.63 (1.52–4.56) **
P for trend						0.07	<0.001	0.001

Notes: * $p < 0.05$; ** $p < 0.01$; *** $p < 0.001$; [a] Complete case analysis; [b] Adjusting for age, sex, perceived family affluence, marital status of biological parents and school clustering; [c] Complete case analysis and excluding students with SHS at home; [d] Adjusting for age, sex, perceived family affluence, marital status of biological parents and school clustering; [e] Complete case analysis and excluding students without family smoking; [f] Adjusting for the number of co-residing smokers at home, age, sex, perceived family affluence, marital status of biological parents and school clustering.

Table S3. Adjusted odds ratio (AOR) of family unhappiness by family smoking and SHS exposure at home (after multiple imputation).

Study Factors	Model 1, N = 1238	Model 2, N = 938 [b]
Number of co-residing smokers at home	AOR (95% CI) [a]	AOR (95% CI) [c]
None	1	1
1	3.13 (2.22–4.40) ***	2.27 (1.51–3.43) ***
2 or more	3.67 (1.97–6.85) ***	3.67 (1.07–12.59) *
Any	3.26 (2.24–4.76) ***	2.53 (1.56–4.11) ***
p for trend	<0.001	0.002
		**Model 3, N = 507 [d]**
SHS exposure at home		AOR (95% CI) [e]
None		1
1–4 days/week		1.37 (0.97–1.94)
5–7 days/week		2.16 (1.31–3.56) **
Any		1.74 (1.21–2.51) **
p for trend		0.003

Notes: * $p < 0.05$; ** $p < 0.01$; *** $p < 0.001$; [a] Adjusting for age, sex, perceived family affluence, marital status of biological parents and school clustering; [b] Excluding students with SHS at home; [c] Adjusting for age, sex, perceived family affluence, marital status of biological parents and school clustering; [d] Excluding students without family smoking; [e] Adjusting for the number of co-residing smokers at home, age, sex, perceived family affluence, marital status of biological parents and school clustering.

Table S4. Adjusted odds ratio (AOR) of tobacco-related unpleasant experience by family smoking and SHS exposure at home (by CEM).

Study Factors	Model 1, N = 962 [a]	Model 2, N = 721 [c]
Number of co-residing smokers at home	AOR (95% CI) [b]	AOR (95% CI) [d]
None	1	1
1	4.83 (3.42–6.81) ***	3.95 (2.55–6.12) ***
2 or more	5.14 (3.27–8.06) ***	5.33 (2.82–10.08) ***
Any	4.90 (3.49–6.87) ***	4.19 (2.87–6.12) ***
p for trend	<0.001	<0.001
		**Model 3, N = 218 [e]**
SHS exposure at home		AOR (95% CI) [f]
None		1
1–4 days/week		1.18 (0.87–1.58)
5–7 days/week		2.21 (0.67–7.30)
Any		1.54 (1.02–2.33) *
p for trend		0.14

Notes: * $p < 0.05$; *** $p < 0.001$; [a] CEM was used to match on sex, age, number of bedrooms at home, perceived family affluence, marital status of biological parents and SHS outside home. Complete case analysis was used; [b] Adjusting for age, number of bedrooms at home, perceived family affluence, SHS outside home and school clustering; [c] After excluding students with SHS at home, CEM was used to match on the same set of covariates in a. Complete case analysis was used; [d] Adjusting for the same set of covariates in b and school clustering; [e] After excluding students without family smoking, CEM was used to match on the number of co-residing smokers at home in addition to the same set of covariates in a. Complete case analysis was used; [f] Adjusting for the number of co-residing smokers at home in addition to the same set of covariates in b and school clustering.

Table S5. Adjusted odds ratio (AOR) of family unhappiness by family smoking and SHS exposure at home (by CEM).

Study Factors	Model 1, N = 974 [a]	Model 2, N = 726 [c]
Number of co-residing smokers at home	AOR (95% CI) [b]	AOR (95% CI) [d]
None	1	1
1	3.05 (2.22–4.18) ***	2.09 (1.27–3.43) **
2 or more	3.45 (1.84–6.49) ***	4.10 (1.81–9.30) **
Any	3.14 (2.37–4.16) ***	2.42 (1.57–3.74) ***
p for trend	<0.001	<0.001
		**Model 3, N = 224 [e]**
SHS exposure at home		AOR (95% CI) [f]
None		1
1–4 days/week		1.75 (0.73–4.17)
5–7 days/week		1.90 (1.02–3.55) *
Any		1.82 (0.92–3.60)
p for trend		0.05

Notes: * $p < 0.05$; ** $p < 0.01$; ***$p < 0.001$; [a] CEM was used to match on sex, age, number of bedrooms at home, perceived family affluence, marital status of biological parents and SHS outside home. Complete case analysis was used; [b] Adjusting for age, number of bedrooms at home, perceived family affluence, SHS outside home and school clustering; [c] After excluding students with SHS at home, CEM was used to match on the same set of covariates in [a]. Complete case analysis was used; [d] Adjusting for the same set of covariates in [b] and school clustering; [e] After excluding students without family smoking, CEM was used to match on the number of co-residing smokers at home in addition to the same set of covariates in [a]. Complete case analysis was used; [f] Adjusting for the number of co-residing smokers at home in addition to the same set of covariates in [b] and school clustering.

References

1 Blackwell, M.; King, G.; Iacus, S.; Porro, G. Cem: Coarsened exact matching in Stata. *Stata J.* **2010**, *9*, 524–546.

2 Iacus, S.M.; King, G.; Porro, G. Causal inference without balance cheching: Coarsened Exact Matching. *Polit. Anal.* **2012**, *20*, 1–24.

3 Ho, D.E.; Imai, K.; King, G.; Stuart, E.A. Matching as nonparametric preprocessing for reducing model dependence in parametric causal inference. *Polit. Anal.* **2005**, *15*, 199–236.

© 2015 by the authors; licensee MDPI, Basel, Switzerland. This article is an open access article distributed under the terms and conditions of the Creative Commons Attribution license (http://creativecommons.org/licenses/by/4.0/).
